# Study on a Rheological Constitutive Model with Yield and Aging Effects for Polyethylene Gas Pipes

**DOI:** 10.3390/polym17162177

**Published:** 2025-08-08

**Authors:** Rui-Hua Yin, Si-Xi Zha, Jun-Qiang Wang, Hui-Qing Lan

**Affiliations:** 1 Key Laboratory of Vehicle Advanced Manufacturing, Measuring and Control Technology, Beijing Jiaotong University, Beijing 100044, China; 24110397@bjtu.edu.cn; 2 School of Mechanical Engineering, Xinjiang University, Ürümqi 830000, China; sxzha@xju.edu.cn; 3 China Special Equipment Inspection and Research Institute (CSEI), Beijing 100029, China

**Keywords:** polyethylene, gas pipe, rheological constitutive model, thermo-oxidative aging

## Abstract

Constitutive models and deformation behaviors for polymer materials have long been complex and are always a hot research focus. As a typical semi-crystalline polymer, polyethylene (PE) gas pipes exhibit pronounced nonlinearity, strain dependence, and time dependence during long-term service. Simple material models fail to capture the scale-dependent characteristics of the PE pipes, resulting in difficulties in accurately describing and simulating their deformation and damage behavior. Currently, some PE gas pipes have entered the mid-to-late stages of service life, so it is necessary to propose a constitutive model representing their complex mechanical behavior for simulation and performance evaluation purposes. Based on results from aging tests, tensile tests, differential scanning calorimetry, and Fourier-transform infrared spectroscopy, this study proposes a method to select a rheological framework and a constitutive model that couples thermo-oxidative aging effects in PE gas pipes. The model is developed within the widely recognized rheological framework and is grounded in continuum mechanics, continuum damage mechanics, and the aging behavior of polymer materials. This method and model are suitable for characterizing the mechanical dependency of PE pipes and demonstrate strong fitting performance. According to the calculation results, the goodness of fit of this constitutive model for the uniaxial tensile test results at the different aging times ranges from 0.982 to 0.999. The findings provide theoretical support for the simulation and service life prediction for PE pipelines.

## 1. Introduction

Polyethylene (PE) pipes are widely used in urban lifeline infrastructure due to their excellent corrosion resistance, high flexibility, and ease of maintenance. However, with the extensive installation and long-term use of PE pipes, related safety issues have become increasingly prominent [1], highlighting the need for accurate material models to characterize their properties in simulation and evaluation processes.

PE is a material with high molecular weight and a complex network structure, and its stress–strain relationship is closely related to its molecular structure, elemental composition, and crosslinking density of molecular chains [2,3,4,5]. Over years of development, two main types of hyperelastic constitutive models have emerged: phenomenological theories based on continuum mechanics and statistical mechanical theories based on molecular structure and conformational entropy changes [6]. All phenomenological theories lack direct physical connections with deformation mechanisms. In contrast, statistical models, which consider materials at the molecular level, can better describe phase transitions. There are two types of statistical constitutive models: those based on Gaussian chain statistics and those using non-Gaussian statistics [7]. Kuhn, based on the assumptions of Gaussian chains and affine deformation, proposed the Affine model. Among the widely applied models is the four-chain model proposed by Flory, a Nobel laureate and renowned polymer physical chemist. James developed the Phantom model to study the elastic deformation of polymers, while Ronce and Allegra proposed the Constrained Junction model [8,9,10,11]. One of the most significant models in the past three decades is the eight-chain model, introduced in 1993 by Professors Arruda and Boyce [12]. This model assumes that macromolecules (also known as chain molecules) are on average located along the diagonal of the unit cell in the principal stretch space. For phenomenological models, materials are generally treated as isotropic, and the strain energy density function is expressed using three strain invariants. Currently, several models are widely used, including: the Neo-Hookean model, Mooney model, Mooney–Rivlin model, Valanis–Landel (VL) model, Ogden model, Yeoh model, Gent model, and Mansouri–Darijani model [13,14,15,16,17,18,19,20]. To date, the development of constitutive models related to polymer elasticity is relatively mature, though the derivational relationships between models remain complex.

In addition to the elastic properties, PE typically exhibits viscoelastic and yield-related behaviors. Regarding viscoelasticity, the modeling has evolved from the early Maxwell model and Kelvin (Kelvin-Voigt) model to three-element and four-element models, and further to the generalized Maxwell and generalized Kelvin models. These models have become well-developed, with many now directly embedded in commercial finite-element software packages. In recent years, to further advance this field, many researchers have introduced mechanical elements involving fractional derivatives to replace traditional dashpots, leading to the development of fractional viscoelastic models [21,22]. Among integral-type constitutive models, a representative example is the Schapery nonlinear viscoelastic model [23], along with a series of specialized models subsequently developed to address specific problems based on it [24]. For viscoplastic constitutive models, Bergstrom et al. [25] developed a hybrid model that incorporates features from the Hasan–Boyce model, Arruda–Boyce model, and the Bergstrom–Boyce model within a plasticity framework. Related ratcheting effects have been described by Drozdov and Christiansen [26], as well as Hassan et al. [27]. As for viscoelastic–viscoplastic constitutive models, in order to simultaneously account for the viscoelastic and viscoplastic deformation of polymer materials under cyclic loading, researchers such as Schapery [28], Drozdov [29], Frank and Brockman [30], Khan and Zhang [31], and Kim and Muliana [32] have developed constitutive models based on experimental studies to describe the creep and relaxation behavior of polymers and polymer-based composites. For elastoviscoplastic models, many studies have adopted phenomenological frameworks to describe the mechanical behavior of semi-crystalline thermoplastics. Le [33], Nikolov and Doghri [34,35], and Dommelen et al. [36] proposed physical inelastic models under finite strain conditions to investigate the large deformation behavior of high-density polyethylene (HDPE) during monotonic loading and unloading.

The rheological model for semi-crystalline thermoplastics (such as PE) was first proposed by Boyce et al. [37], who described the deformation mechanism using a network resistance approach, capturing polymer-chain sliding and stretching as deformation modes that act in parallel with intermolecular resistance to account for plasticity. Subsequently, based on micromechanical modeling, researchers such as Ahzi et al. [38], Makradi et al. [39], Makki et al. [40,41], Ayoub et al. [42], Liang [43], Zhu [44], and PHao [45] further refined the resistance between molecular chains and the resistance between molecular networks, incorporating the complex micromechanical behavior of the amorphous and crystalline phases. The current rheological model has broken through the limitations of traditional models and can accurately predict the stress–strain behavior of polymer materials. However, research on material aging degradation has long been lacking. From the perspective of aging and degradation, the long-term time-dependent aging characteristics of polymers are difficult to reflect in the aforementioned models. Some researchers have considered the effects of aging on constitutive behavior, but such studies remain limited and relatively superficial. For instance, Belbachir et al. [46] incorporated the effects of ultraviolet aging into a physical elastic–viscoplastic model to capture the mechanical degradation behavior of PLA. Ayoub et al. [47] employed a physical viscoelastic–viscoplastic approach to simulate the mechanical and fracture responses of semi-crystalline low-density polyethylene films under accelerated UV aging.

Overall, this paper proposes a viscoelastic–viscoplastic rheological constitutive model based on thermal-oxidative aging parameters. This model integrates rheological theory and continuum mechanics theory, while accounting for the accelerated degradation process of PE gas pipelines under thermal-oxidative aging. It incorporates crystallinity data obtained from DSC and carbonyl indices measured by FTIR under different aging conditions. This approach provides a novel methodology for constructing constitutive models considering aging degradation.

## 2. Theories

### 2.1. Rheological Model Framework

**Figure 1:**
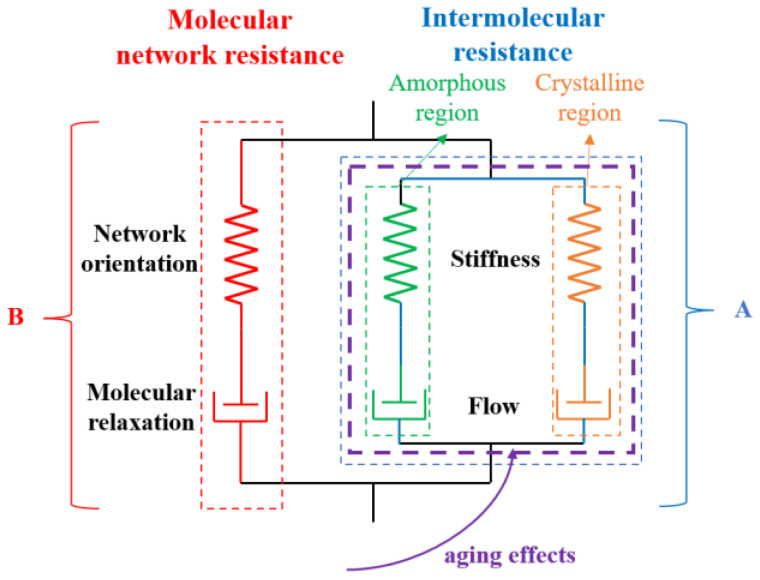
The frame of the constitutive model in the paper.

In this study, the basic framework of the rheological structure is illustrated in Figure 1. It involves two independent fundamental mechanisms: (A) resistance caused by intermolecular interactions between adjacent polymer segments, and (B) network forces resulting from molecular alignment and relaxation. These two mechanisms operate in parallel. The intermolecular interactions contribute to the initial stiffness in the stress–strain response, as well as the rate dependence and temperature sensitivity of the initial flow behavior; the network interactions, on the other hand, lead to anisotropic hardening behavior in PE materials due to molecular orientation under tensile loading [38,39,43]. Network A comprises two parallel network resistance branches, representing the amorphous and crystalline phases, respectively. Network B consists of a linear spring in series with a dashpot, and a nonlinear spring modeled by the eight-chain model in series with a nonlinear dashpot, forming a branch that represents network resistance. It is assumed that thermo-oxidative aging under internal pressure primarily affects the intermolecular resistance in the PE pipe material, which is reflected in changes to the elastic modulus of the intermolecular spring and the viscous flow rate. The total Cauchy stress T in the PE pipe material is equal to the tensor sum of the stresses in Network A $T_{A}$ and Network B $T_{B}$. Since the intermolecular resistance and network resistance are arranged in parallel, the deformation gradients of each branch $F_{A}$ and $F_{B}$ are equal to the total deformation gradient F.

(1) $$\left\{\begin{array}{l} T=T_{A}+T_{B}\\ F=F_{A}=F_{B} \end{array}\right.,$$

Furthermore, by decomposing the multiplicative decomposition of the deformation gradient tensor into elastic $F^{e}$ and plastic $F^{p}$ deformation gradient tensors, we obtain $F=F^{e}F^{p}$. The velocity gradient is defined as $L=\dot{F}F^{-1}$ and can be further additively decomposed into L=D+W, where D represents the deformation rate and W is the spin tensor. Simultaneously, the velocity gradient tensor can also be decomposed into the sum of elastic and plastic velocity gradients: $L=L^{e}+L^{p}$. Based on the studies by Boyce, Ayoub, and others, it is assumed that plastic flow is incompressible and irrotational, with the plastic deformation gradient expressed as ${\dot{F}}^{p}=F^{e^{-1}}D^{p}F^{e}F^{p}=F^{e^{-1}}D^{p}F$. Meanwhile, Ayoub and Cundiff et al. have demonstrated the thermodynamic consistency of this method.

### 2.2. Network A: Intermolecular Forces

Network A consists of two branches, representing the crystalline and amorphous regions, respectively. Each branch is composed of a linear spring and a nonlinear dashpot, where the initial stiffness response arises from intermolecular resistance, followed by intermolecular flow, representing yield behavior and strain-rate dependence. The most basic form of elastic theory is isotropic elasticity, whose constitutive relation is expressed as follows:

(2) $$T_{A}^{i}=\frac{1}{J_{A}^{ei}}C_{A}^{ei}\ln(\lambda^{i})\;\left(i=a,c\right),$$

where i = 1, 2 represents Branch 1 and Branch 2; $J_{A}=detF_{A}^{e}$ denotes the volumetric change; $\ln(\lambda_{i})$ is the Hencky strain; and $C_{A}^{e}$ is the fourth-order elastic stiffness tensor, expressed in index notation as:

(3) $${\left({\mathbb{C}}_{A}^{ei}\right)}_{jikl}=\frac{E_{A}^{i}}{2\left(1+v_{A}^{i}\right)}\left[\left(\delta_{ik}\delta_{jl}+\delta_{il}\delta_{jk}\right)+\frac{2v_{A}^{i}}{1-2v_{A}^{i}}\delta_{ij}\delta_{M}\right],$$

where δ is the Kronecker delta, $E_{A}^{i}$ and $v_{A}^{i}$ are the elastic modulus and Poisson’s ratio of the linear spring in Network A, respectively. The viscous deformation of Network A can be calculated using the following equation:

(4) $${\tilde{D}}_{A}^{pi}={\dot{\gamma }}_{A}^{pi}\;N_{A}^{pi},$$

where $\tau^{i}={\left\|\mathrm{dev}\left[T_{A}^{i}\right]\right\|}_{F}=\sqrt{\mathrm{tr}\left[T_{A}^{iT}T_{A}^{i^{\prime }}\right]}$ is the effective stress driving viscous flow; $\|\cdot {\|}_{F}$ denotes the Frobenius norm; and the direction of viscous flow is expressed as:

(5) $$N_{A}^{pi}=\frac{\mathrm{dev}\left[T_{A}^{i}\right]}{\tau^{i}}=\frac{\mathrm{dev}\left[T_{A}^{i}\right]}{{\left\|\mathrm{dev}\left[T_{A}^{i}\right]\right\|}_{F}},$$

The time derivative of the plastic deformation gradient for a given branch of Network A is expressed as:

(6) $${\dot{F}}_{A}^{Pi}={\dot{\gamma }}_{A}^{Pi}{\left(F_{A}^{ei}\right)}^{-1}\frac{\mathrm{dev}\left[T_{A}^{i}\right]}{{\left\|\mathrm{dev}\left[T_{A}^{i}\right]\right\|}_{F}}F_{A}^{ei}F_{A}^{pi},$$

The time-derivative equation of the deformation gradient described above is purely kinematics-based. Now, only the effective creep rate (viscous flow rate) needs to be determined to compute the deformation gradient of this network. This study adopts the Exponential Energy-Activated Flow model because it can effectively characterize the yield evolution process. The specific expression of this flow model is given as follows:

(7) $${\dot{\gamma }}_{A}^{pi}={\dot{\gamma }}_{0}^{i}\exp\left[-\frac{∆G}{k\theta }\left(1-\frac{\tau }{f_{p}f_{\varepsilon^{p}}f_{\theta }{\hat{\tau }}_{A}^{i}}\right)\right],$$

where ${\dot{\gamma }}_{0}$ is a material parameter referred to as the pre-exponential factor (or attempt frequency) with units of $s^{-1}$; ${\hat{\tau }}_{A}$ is the shear flow resistance; ∆G is the activation energy; k is the Boltzmann constant; θ is the absolute temperature; f is the correction factor for shear flow resistance. This study does not consider the pressure correction factor, focusing only on yield evolution and temperature correction factors. Yield evolution typically describes how the yield stress of glassy polymers increases with accumulated plastic strain or strain softening after initial yielding. The flow resistance in the flow model can evolve based on the applied equivalent plastic strain:

(8) $$\varepsilon^{pi}=\sqrt{\frac{2}{9}\left[{\left(\varepsilon_{1}^{pi}-\varepsilon_{2}^{pi}\right)}^{2}+{\left(\varepsilon_{2}^{pi}-\varepsilon_{3}^{pi}\right)}^{2}+{\left(\varepsilon_{3}^{pi}-\varepsilon_{1}^{pi}\right)}^{2}\right]},$$

A linear rate evolution under plastic strain is adopted:

(9) $$f_{g^{p}}^{i}=f_{1}\left(\varepsilon^{pi}\right)+g\cdot \left(\lambda_{\mathrm{chain}}^{i}-1\right),$$

where $\lambda_{\mathrm{chain}}^{i}=\sqrt{tr[b^{pi}]/3}$ is the chain stretch and the shear resistance $f_{1}\left(\varepsilon^{pi}\right)$ can be expressed as:

(10) $${\dot{f}}_{1}=h\times \left[1-\frac{f_{0}^{i}}{f_{\max}^{i}}\right]\times {\dot{\gamma }}_{A}^{pi},$$

where h is the yield drop slope relative to plastic strain; $f_{\max}^{i}$ is the final value; $f_{0}^{i}$ is the initial value. For the temperature correction factor, the Arrhenius-type correction is adopted:

(11) $$f_{\theta }=\exp\left[-\frac{E}{R}\cdot \left(\frac{1}{\theta }-\frac{1}{\theta_{0}}\right)\right],$$

where E/R is the ratio of activation energy to the gas constant; $\theta_{0}$ is the reference temperature; θ is the current temperature; if θ= $\theta_{0}$ and $f_{0}=1$, this correction factor is disregarded (the tensile testing is performed at a room temperature 293 K).

### 2.3. Network B: Molecular Network Alignment

Network B consists of parallel branches formed by a nonlinear spring and a nonlinear dashpot. The nonlinear spring describes the anisotropic strain hardening resulting from molecular network stretching, while the nonlinear dashpot accounts for the relaxation process caused by molecular “chain reptation”. These two parallel branches represent the crystalline phase network and the amorphous phase network, respectively. Throughout the deformation process, stretching and relaxation mechanisms “compete” with each other. In fact, molecular orientation increases stress through deformation, while molecular relaxation can reduce stress by adjusting components of the applied deformation. The Cauchy stress of Network B is determined using an eight-chain model based on non-Gaussian statistical theory (detailed in Appendix A.1.), with its specific expression given as follows:

(12) $$T_{B}=\frac{\mu_{B}}{J_{B}^{e}\bar{\lambda_{B}^{e*}}}\frac{L^{-1}\left(\frac{\bar{\lambda_{B}^{e*}}}{\lambda^{\mathrm{lock}}}\right)}{L^{-1}\left(\frac{1}{\lambda^{\mathrm{lock}}}\right)}\mathrm{dev}\left[b_{B}^{e*}\right]\;+\;\kappa \left(J_{\mathrm{BA}}^{e*}\;-\;1\right)I,$$

where $J_{B}^{e}=\det[F_{B}^{e}]$ is the Jacobian determinant of the elastic deformation gradient, representing volume change; $\mu_{B}$ is the initial shear modulus; $b_{B}^{*}=(J_{B}^{e}{)}^{-2/3}F_{B}^{*T}(F_{B}^{*})$ is the left Cauchy–Green strain tensor; $\bar{\lambda_{B}^{e^{*}}}={\left(\mathrm{tr}[b_{B}^{e}]/3\right)}^{1/2}$ is the equivalent chain stretch based on the eight-chain model’s topological assumption; $L^{-1}(x)$ is the inverse Langevin function; L(x)=coth(x)−1/x; $\lambda^{\mathrm{lok}}\equiv nl$ is the locking (limiting) stretch, representing the maximum (fully extended) elongation a PE molecule can achieve; κ is the bulk modulus. For incompressible cases, terms containing the bulk modulus can be neglected. The viscous deformation of Network B can similarly be calculated using the following equation:

(13) $${\tilde{D}}_{B}^{p}={\dot{\gamma }}_{B}^{P}N_{B}^{p},\;N_{A}^{p}=\frac{\mathrm{dev}\left[T_{B}\right]}{\tau }=\frac{\mathrm{dev}\left[T_{B}\right]}{{\left\|\mathrm{dev}\left[T_{B}\right]\right\|}_{F}},$$

Similarly, the time derivative of the plastic deformation gradient $F_{B}^{p}$ for Network B is expressed as:

(14) $${\dot{F}}_{B}^{P}={\dot{\gamma }}_{B}^{P}{\left(F_{B}^{e}\right)}^{-1}\frac{\mathrm{dev}\left[T_{B}\right]}{{\left\|\mathrm{dev}\left[T_{B}\right]\right\|}_{F}}F_{B}^{e}F_{B}^{p},$$

The effective creep rate of Network B adopts the Bergström–Boyce (B-B) flow model, which is explained in Appendix A.2.

### 2.4. Network A: Aging Effect

This study does not consider specific thermo-oxidative aging reaction equations, such as how chemical mechanisms such as radical chain reactions functionally influence the constitutive model (material volume shrinkage induced by chemical reactions is neglected). Instead, we focus solely on the quantitative evolution of material parameters within the constitutive framework to incorporate thermo-oxidative aging effects. Based on experimental findings [39], aging primarily affects intermolecular interactions [44,46,47]. The aging process significantly alters key performance parameters of PE pipe materials, including elastic modulus, yield strength, elongation at break, and crystallinity.

Aging coefficient normalization: To characterize the evolution of mechanical properties in PE pipe materials under thermo-oxidative aging, crystallinity and carbonyl index are selected as representative aging metrics. The thermo-oxidative aging parameter is defined as:

(15) $$\beta =\omega_{1}\frac{∆CI}{CI_{\mathrm{crit}}}+\omega_{2}\frac{∆\chi_{C}}{\chi_{\mathrm{crit}}}=\omega_{1}\frac{CI\left(t\right)-CI_{\min}}{CI_{\max}-CI_{\min}}+\omega_{2}\frac{\chi \left(t\right)-\chi_{\min}}{\chi_{\max}-\chi_{\min}},$$

where ω is the weighting factor; CI(t) and χ(t) are the actual aged carbonyl index and crystallinity, respectively; $CI_{\mathrm{crit}}$ and $\chi_{\mathrm{crit}}$ are the reference carbonyl index and crystallinity.

Due to the elevated temperatures typically employed in accelerated aging tests, diffusion-limited oxidation (DLO) [48,49,50,51] behavior occurs, leading to spatially non-uniform distributions of crystallinity and carbonyl index across the material, particularly along the pipe wall thickness. Thus, the aging parameter in the above equation is assumed to represent the average value along the pipe wall. Furthermore, since both crystallinity and carbonyl index serve as critical indicators of aging effects while exhibiting certain similarities and differences, a weighted formula is adopted to define the aging parameter. The weight distribution is determined using the entropy weight method, yielding $\omega_{1}$ = 0.5290 and $\omega_{2}\;$= 0.4710. The detailed evaluation procedure is provided in Appendix A.3. During the initial aging stages, reactions in the amorphous regions are more pronounced, whereas crystalline region degradation dominates long-term aging effects on overall performance especially in high-crystallinity materials. Notably, studies by Bédoui and Ayoub et al. [52,53] and subsequent parameter fitting revealed that although the elastic moduli of crystalline and amorphous regions differ significantly, the normalized moduli exhibit nearly identical trends with aging coefficients. Therefore, it assumes each region has the same aging state, i.e., their normalized parameters follow the same aging-coefficient-dependent trends.

The mechanical property parameters (elastic modulus, yield strength, and elongation at break), crystallinity, and carbonyl index were extracted and plotted to create parameter evolution curves. A Boltzmann-type function was selected for fitting, establishing quantitative relationships (normalized representation) between the key mechanical properties and the thermo-oxidative aging parameter. Meanwhile, the total Cauchy stress of Network A was defined with reference to crystallinity as follows:

(16) $$E_{A}\left(E_{A}^{i}\right)=E_{A0}\left(E_{A0}^{i}\right)\left(\frac{c_{1}-c_{2}}{1+e^{\beta /d\beta }}+c_{2}\right),$$

(17) $$\sigma_{yA}\left(\sigma_{yA}^{i}\right)=\sigma_{yA0}\left(\sigma_{yA0}^{i}\right)\left(\frac{c_{3}-c_{4}}{1+e^{\beta /d\beta }}+c_{4}\right),$$

(18) $$\varepsilon_{fA}\left(\varepsilon_{fA}^{i}\right)=\varepsilon_{fA0}\left(\varepsilon_{fA0}^{i}\right)\left(\frac{c_{5}-c_{6}}{1+e^{\beta /d\beta }}+c_{6}\right),$$

(19) $$T_{A}=\chi_{C}T_{A}^{c}+\left(1-\chi_{C}\right)T_{A}^{a},$$

where $E_{A0}^{i}$, $\sigma_{yA0}^{i}$, and $\varepsilon_{fA0}^{i}$ are the initial values; $c_{i}$ is the fitting parameter.

### 2.5. Network A: Yield Evolution

The yield point of a material marks the transition from purely elastic to elastoplastic behavior. For PE pipes, the definition of yield strength or yield stress remains subject to varying interpretations, and no universally accepted standard currently exists to determine this transition. In the case of polymers, the strain offset used to define yield is widely debated, typically ranging from 0.3% to 2% [54]. In this study, a 0.3% strain offset is adopted to determine the yield stress.

In the unaged state, the pre-exponential factor ${\dot{\gamma }}_{0}$ is typically set to $10^{4}s^{-1}$[43]. Based on the following transformation of Equation (7), the activation energy ∆G and initial shear strength $S_{y}$ can be determined:

(20) $$\tau^{i}=\frac{k\theta f_{\varepsilon^{p}}{\hat{\tau }}_{A}^{i}}{∆G}\ln\left(\frac{{\dot{\gamma }}_{A}^{P}}{{\dot{\gamma }}_{0}}\right)+f_{\varepsilon^{p}}{\hat{\tau }}_{A}^{i},$$

According to Equation (9) and B-5, at the onset of tensile loading, $\lambda_{\mathrm{chain}}=1$, ${\dot{\gamma }}_{A}^{P}=0$ and $f_{\varepsilon^{p}}=f_{0}$.

(21) $$\tau^{i}=\frac{k\theta f_{0}{\hat{\tau }}_{i}^{A}}{∆G}\ln\left(\frac{{\dot{\gamma }}_{A}^{P}}{{\dot{\gamma }}_{0}}\right)+f_{0}{\hat{\tau }}_{A}^{i},$$

where the plastic shear strain rate ${\dot{\gamma }}_{A}^{pi}$ is approximately $\sqrt{3}\dot{\varepsilon }$. Using data from different strain rates and initial shear flow stresses, ∆G and $S_{y}$ are determined via least-squares fitting. Dividing ∆G by the test temperature yields the activation volume. The initial shear stress $\tau^{i}$ is roughly 1/$\sqrt{3}$ the tensile strength measured in uniaxial tests at corresponding strain rates, and is nearly equivalent to the yield strength. By incorporating parameter h, Equation 10 can be reformulated as follows for calculation:

(22) $$h={\left[1-\frac{f_{0}}{f_{\max}}\right]}^{-1}\times \frac{{\dot{f}}_{1}}{{\dot{\gamma }}_{A}^{P}}\approx {\left[1-\frac{f_{0}}{f_{\max}}\right]}^{-1}\times \frac{∆f}{∆\gamma_{A}^{P}}=\frac{f_{\max}}{∆\gamma_{A}^{P}},$$

For the Network B, the primary parameter that needs to be determined is the shear modulus $\mu_{B}$.

(23) $$\mu_{B}=\frac{Nk\theta }{3}\lambda^{\mathrm{lock}-1}\left(\frac{1}{\lambda^{\mathrm{lock}}}\right),$$

where N is the average number of chains per unit volume, k is the Boltzmann constant, and θ is the absolute temperature.

With Equations (17) and (21), the variation patterns of activation energy ∆G and initial shear strength $S_{y}$ with respect to the thermo-oxidative aging factor for PE80 and PE100 pipes can be obtained. Similarly, the normalized variation of ∆G and $S_{y}$ for PE80 and PE100 pipe materials with the thermo-oxidative aging factor can also be determined using Equation (24).

(24) $$\left\{\begin{array}{l} ∆G=∆G_{0}\left(\frac{A_{1}-A_{2}}{1+e^{\beta /d\beta }}+A_{2}\right)\\ S_{y}=S_{y0}\left(\frac{A_{3}-A_{4}}{1+e^{\beta /d\beta }}+A_{4}\right) \end{array}\right.,$$

Ayoub et al. [40,48] proposed empirical Equations (25) and (26) for estimating the initial values of shear strength $S_{y}$ and activation energy ∆G in polyethylene with varying crystallinity levels. Based on experimental data of elastic stiffness from Solvay’s PE materials [49], the initial value of elastic modulus can be determined.

(25) $$S_{y}^{i}=5\chi +0.055,$$

(26) $$∆G^{i}=4\times 10^{-21}+7\times 10^{-20}\exp\left(-2.31\chi \right),$$

## 3. Experiment

Two grades of PE pipes (PE80 and PE100) for thermal-oxidative aging tests were provided by Nansu Plastic Products (Shenzhen, China) Co., Ltd. Table 1 lists the basic material properties of the two PE pipes under unaged conditions. In this paper, the ratio of pipe diameter to wall thickness, namely the Standard Dimension Ratio (SDR), was selected as 11. Both PE pipe materials were added with 0.1% by weight of Irganox 1010 and 0.1% of Irgafos 168 antioxidants and stabilizers. Additionally, the PE80 material contains 5% by weight of carbon black. For specific FTIR and DSC test results and procedures, please refer to reference [55]; the authors of the paper only carried out tensile tests in this paper and used relevant test data to construct a numerical model.

To investigate the thermo-oxidative aging of PE pipes under accelerated conditions, aging tests were conducted using a specialized aging device [55]. Notably, both the external and internal surfaces of the PE pipes were exposed to air. The pipes were placed in an oven with adjustable temperature control to 353 K. For precise control of internal pressure in the PE pipes, a custom control program was developed to maintain pressure within 0.01 MPa of the target value (0.4 ± 0.01 MPa). Through coordinated operation of the pressure sensors and solenoid valves, a stable pipe pressure was achieved, with the maximum adjustable pressure range set at 1.0 MPa. Post-aging characterization involved uniaxial tensile tests, differential scanning calorimetry (DSC) and Fourier-transform infrared spectroscopy (FTIR). The monotonic tensile tests were conducted using an electronic universal testing machine (MTS CMT4000) at 23 ± 2 °C. Standard dumbbell-shaped pipe specimens were stretched at a rate of 50 mm/min in accordance with ISO 6259-3 [56]. These dumbbell-shaped samples were machined from intact pipe specimens using a CNC lathe.

Antioxidant capacity testing was performed on samples using a DSC 200F3 differential scanning calorimeter, with measurements including oxidation onset temperature (OOT) and oxidation induction temperature (OIT). To determine the OOT, samples were heated continuously from 25 °C to 300 °C at 20 °C/min in a pure oxygen atmosphere until reaching the oxidation onset temperature [57]. The OOT value was calculated based on a 0.2 mW baseline shift [58]. For OIT measurement according to ISO 11357-6 [59], samples were first heated to 200 °C under 50 mL/min nitrogen flow, held isothermally for several minutes, then exposed to 50 mL/min pure oxygen while monitoring exothermic behavior. Crystallinity for Equation (15) was obtained through this work.

To characterize the changes in mechanical properties during the aging process through carbonyl index, tests were conducted using a Bruker VERTEX 70 Fourier-transform infrared spectrometer. At each aging interval, degradation of PE pipeline material was assessed through 30 repeated scans at 4 cm^−1^ spectral resolution. The 400–4000 cm^−1^ spectral range was employed to analyze functional group alterations in pipeline samples resulting from thermo-oxidative aging in heated air [60]. Finally, the carbonyl index for Equation (15) was calculated by dividing the peak area of the carbonyl peak (C=O) of 1650~1850 cm^−1^ by the peak area of the methylene peak (CH_2_) of 1330~1500 cm^−1^.

**Figure 2:**
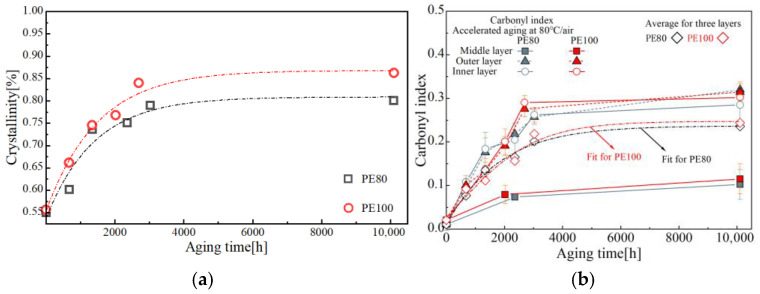
Variations in (**a**) crystallinity and (**b**) carbonyl index with the aging time.

**Figure 3:**
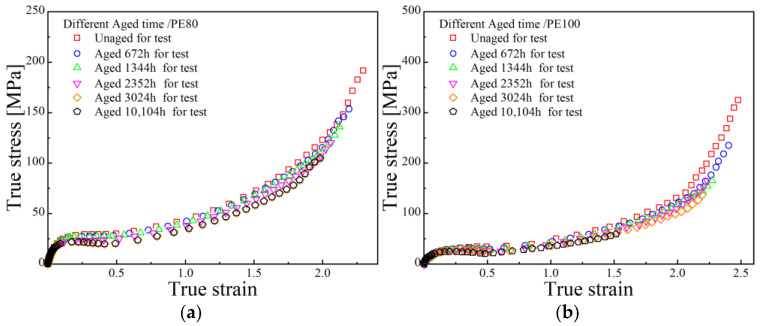
True stress–strain curves at different aging times (**a**) PE80 and (**b**) PE100.

Based on the aforementioned experiments, the evolution of crystallinity and carbonyl index with aging time are presented in Figure 2. Fundamental mechanical parameters were extracted from the engineering and true stress–strain curves shown in Figure 3.

## 4. Results

**Figure 4:**
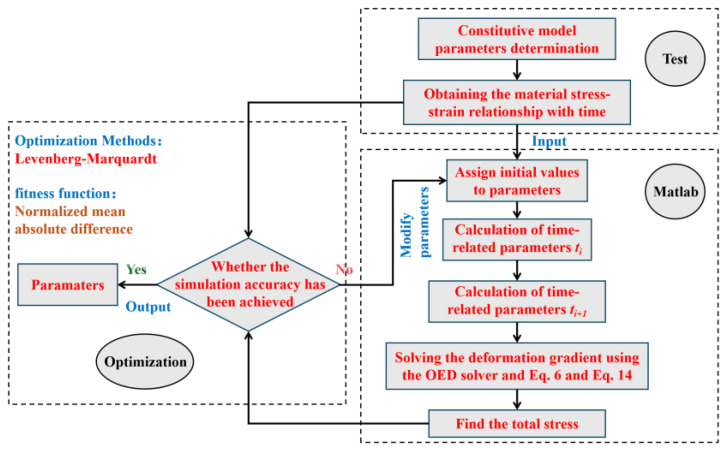
Numerical realization of the constitutive model and parameter fitting.

As discussed in Section 2, the proposed material constitutive model is formulated through a system of differential tensor equations that require solving at each increment. To numerically solve this system of equations, the following iterative method can be employed, with a computational workflow illustrated in Figure 4. The constitutive model incorporates a total of 21 parameters, with the temperature θ set at 293 K. Under the assumption of incompressibility, the Jacobian determinant equals 1 [61]. Consequently, regardless of the bulk modulus value, stress terms containing the bulk modulus vanish and therefore do not affect the final computational results. The shear modulus $\mu_{B}$ and ultimate elongation $\lambda^{lock}$ of MDPE and HDPE are generally around 12 MPa and 5 MPa, respectively (assumed values). The strain adjustment coefficient ξ is typically taken as 0.01. The shear flow index m and strain index C have little impact on the results and are usually set to 3 and −0.5, respectively. The material parameters ${\hat{\tau }}_{B}$, $f_{0}$, $f_{\max}$, g, and n are determined by fitting the tensile test data at quasi-static. The remaining parameters are obtained through the calculated theories and methods above. The proposed material constitutive model is defined by a system of differential equations requiring solution at each increment. To solve this system, the iterative method presented in the flowchart of Figure 4 is employed, Levenberg–Marquardt (LM) methods and normalized mean absolute difference error (NMAD) methods are used for this optimization [62]. The assumed parameters are listed in Table 1; the parameters after fitting are listed in Table 2, Appendix A.4. shows the details for LM with NMAD method.

1. Solve for the relevant parameter values at time $t_{i}$, including:
  Deformation gradient: F
  State variables: $F_{A}^{P1}$, $F_{A}^{P2}$, $F_{B}^{P}$, $f_{1}$
2. Solve for the relevant parameter values at time $t_{i+1}$:
  Deformation gradient: F
3. Use an ODE45 solver and Equation (6) to solve for $F_{A}^{P1}$ and $F_{A}^{P2}$ at time $t_{i+1}$.
4. Use an ODE45 solver and Equation (14) to solve for ${\dot{F}}_{B}^{P}$ at time $t_{i+1}$.
5. Calculate the total stress at time $t_{i+1}$ using Equation (1).
6. Enter the strain value ε, experimentally measured stress value (in uniaxial test) $\dot{T}$, and initial parameter guesses, set the initial damping factor to 0.01, the maximum number of iterations to 30,000, and the convergence threshold to 1 $\times \;10^{-10}$.
7. Calculate the sum of squared residuals S between the experimental stress and the computed stress.
8. Calculate the LM update step $h_{\mathrm{LM}}$ and update all material parameters.
9. Adaptive adjustment of damping factor and NMAD parameter evaluation.
10. Output the optimal parameters.

**Figure 5:**
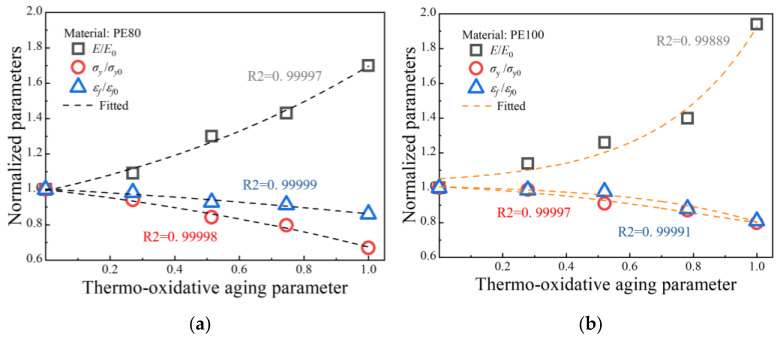
Relationship between thermo-oxidative aging parameters and normalized parameters (**a**) PE80 and (**b**) PE100.

**Figure 6:**
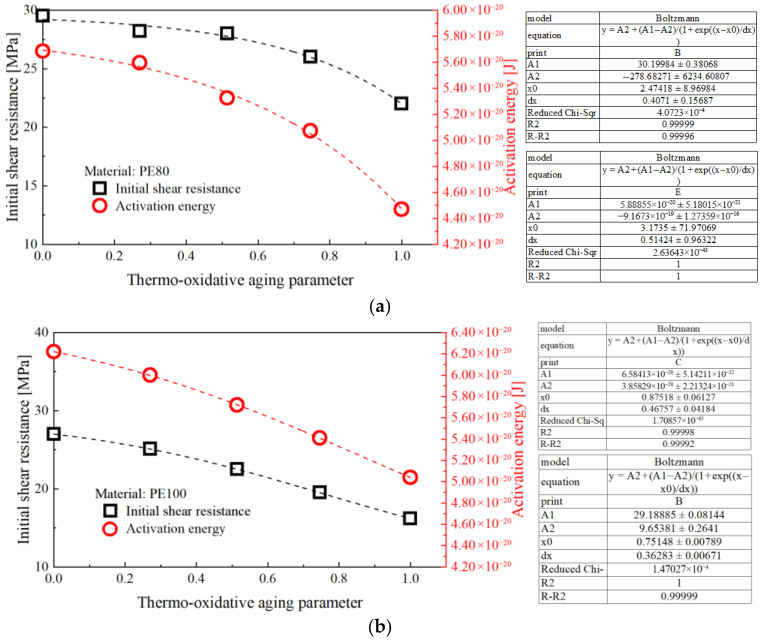
Relationship between initial shear strength, activation energy, and thermo-oxidative aging parameters (**a**) PE80 and (**b**) PE100.

Equations (16)–(18) and (24) establish a Boltzmann-based empirical relationship between thermo-oxidative aging parameters and mechanical properties, highlighting the impact of thermo-oxidative aging on the elastic modulus E, initial shear strength $S_{y}$ and activation energy ∆G of PE pipes [47]. The mechanical property parameters, crystallinity, and carbonyl index obtained in previous sections were extracted and plotted as parameter evolution diagrams in Figure 5 and Figure 6. By fitting these data with the Boltzmann function, quantitative relationships between the main mechanical property parameters and thermo-oxidative aging parameters were established. Once these parameters are known, the parameters related to thermo-oxidative aging can then be identified. Figure 5 shows the relationship between thermo-oxidative aging parameters and normalized parameters and Table 3 and Table 4 show the fitting parameters for PE80 and PE100. Figure 6 shows the relationship between initial shear strength, activation energy, and thermo-oxidative aging parameters and Table 5 and Table 6 show the fitting parameters for PE80 and PE100.

**Figure 7:**
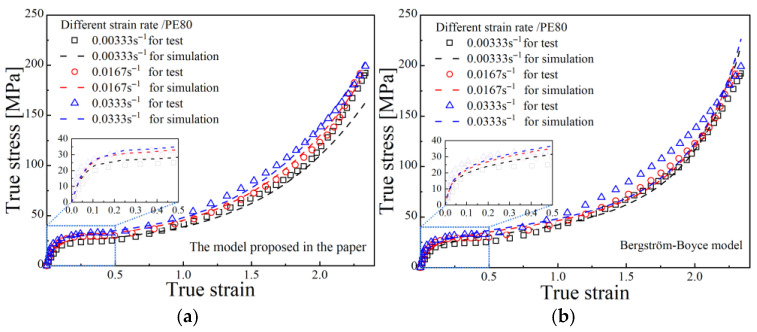

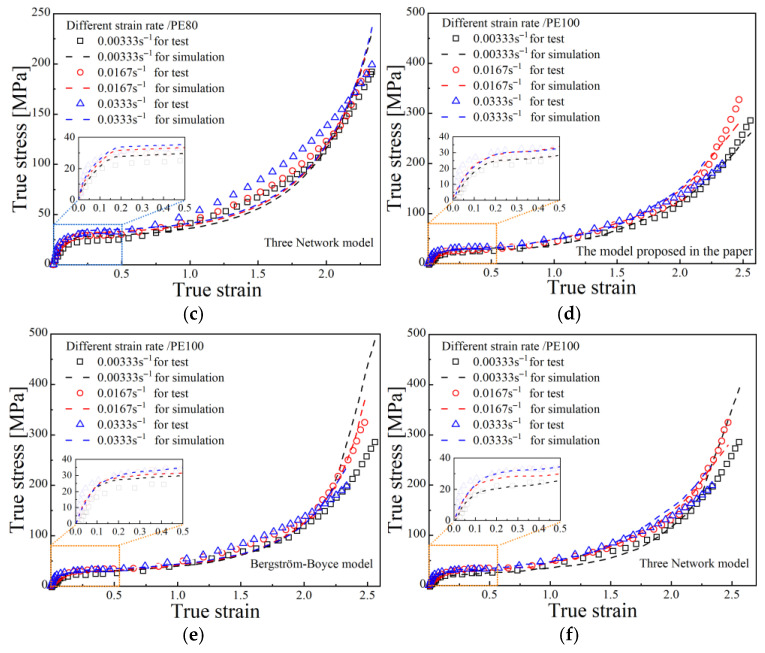
Fitting results of the three models under different strain rates; (**a**) PE80 for proposed model; (**b**) PE80 for the B-B model; (**c**) PE80 for the TNW model; (**d**) PE100 for the proposed model; (**e**) PE100 for the B-B model; (**f**) PE100 for the TNW model.

**Figure 8:**
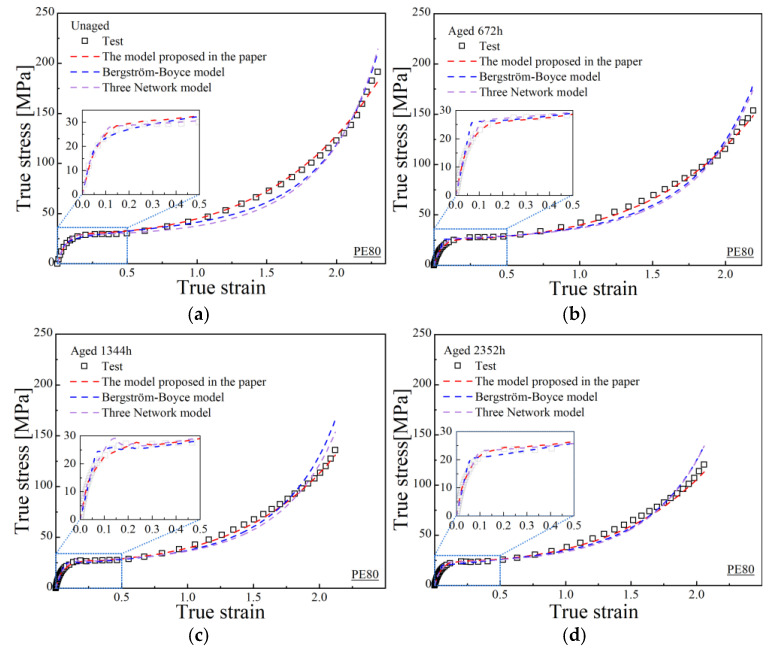

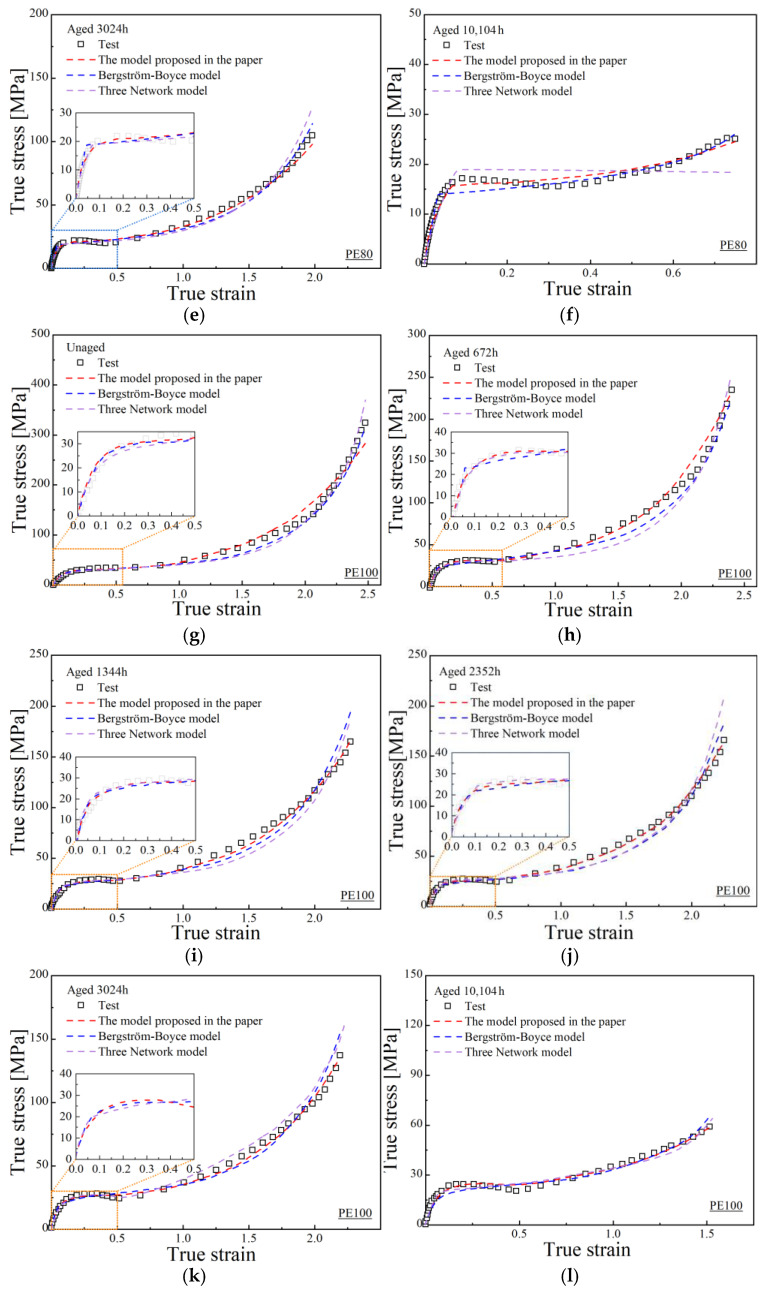
Fitting results of the three models at different aging times for quasi-static test; (**a**) unaged PE80; (**b**) 672 h for PE80; (**c**) 1344 h for PE80; (**d**) 2352 h for PE80; (**e**) 3024 h for PE80; (**f**) 10,104 h for PE80; (**g**) unaged PE100; (**h**) 672 h for PE100; (**i**) 1344 h for PE100; (**j**) 2352 h for PE100; (**k**) 3024 h for PE100; (**l**) 10,104 h for PE100.

Figure 7 first presents the fitting performance of true stress–strain curves for PE80 under different strain rates, comparing the proposed model in this study with the BB model and the TNM. Figure 8 illustrates the fitting results for quasi-static true stress–strain curves at different aging times, also comparing the proposed model with the BB model and the TNM. Table 7 and Table 8 shows the relevant goodness of fit for PE80 and PE100 pipes.

## 5. Conclusions

1. Based on uniaxial tensile test results of two types of PE air pipe dumbbell-shaped specimens under thermo-oxidative aging, and considering the carbonyl index and crystallinity as thermo-oxidative aging parameters, an elastic–viscoplastic constitutive model incorporating both thermo-oxidative aging effects and yield behavior was developed within a finite deformation framework.

2. The model defines carbonyl index and crystallinity as normalized aging parameters for the first time, and demonstrates the temporal evolution of partial constitutive model parameters by fitting the Boltzmann equation

3. Compared to the B-B model and TNM model, this model not only achieves higher prediction accuracy across various test results, the overall goodness-of-fit exceeds 0.982, but more importantly, incorporates variations in material parameters during thermo-oxidative aging thereby significantly enhancing the model’s microscopic interpretability.

4. It should be noted that, like the B-B and TNM models, the current model, which is based on finite deformation theory, calculates stress from strain. Future work will develop a finite-element (FE) simulation model by referencing the methods of Tømmernes and Liang [63,64], and incorporating aging effects into simulation models, such as by developing a UEL (User Element) in ABAQUS, presents a viable approach [65].

## Figures and Tables

**Table 1:** Assumed parameters for unaged PE.

Parameters	Symbols	Unit	PE
Absolute temperature	θ	K	293
Amorphous elastic modulus	$E^{a}$	MPa	2
Crystalline elastic modulus	$E^{c}$	MPa	200
Amorphous and crystalline pre-exponential factor	${\dot{\gamma }}_{0}$	s^−1^	100
Crystalline activation energy	$∆G^{a}$	J	1 × 10^−20^
Amorphous activation energy	$∆G^{c}$	J	1 × 10^−19^
Amorphous initial shear resistance	$f_{0}$	-	0.1
Amorphous flow shear resistance	${\hat{\tau }}_{A}^{a}$	MPa	1
Crystalline flow shear resistance	${\hat{\tau }}_{A}^{c}$	MPa	10
Amorphous final shear resistance	$f_{\max}$	-	5
Crystalline hardening soften slope	$h^{c}$	-	20
Amorphous hardening soften slope	$h^{a}$	-	2
Amorphous and crystalline interaction coefficient	g	-	1
Amorphous and crystalline interaction coefficient	n	-	1
Shear modulus	$\mu_{B}$	MPa	12
Maximum (fully extended) stretch	$\lambda_{\mathrm{lock}}$	-	5
Bulk modulus	κ	MPa	500
Adjustment coefficient	ξ	-	0.01
Strain factor	C	-	−1
Flow shear resistance	${\hat{\tau }}_{B}$	MPa	1
Flow shear modulus	m	-	1

**Table 2:** Final material constant for unaged PE80 and PE100.

Parameters	Symbols	Unit	PE80	PE100
Absolute temperature	θ	K	273	273
Amorphous elastic modulus	$E^{a}$	MPa	3.7	3.75
Crystalline elastic modulus	$E^{c}$	MPa	372	375
Amorphous and crystalline pre-exponential factor	${\dot{\gamma }}_{0}$	s^−1^	104	104
Crystalline activation energy	$∆G^{a}$	J	2.15 × 10^−20^	2.55 × 10^−20^
Amorphous activation energy	$∆G^{c}$	J	1.01 × 10^−19^	1.04 × 10^−19^
Amorphous initial shear resistance	$f_{0}$	-	1	1
Amorphous flow shear resistance	${\hat{\tau }}_{A}^{a}$	MPa	0.21	0.20
crystalline flow shear resistance	${\hat{\tau }}_{A}^{c}$	MPa	29	27
Amorphous final shear resistance	$f_{\max}$	-	2	2
Crystalline hardening soften slope	$h^{c}$	-	69	69
Amorphous hardening soften slope	$h^{a}$	-	0.6	0.6
Amorphous and crystalline interaction coefficient	g	-	1.1	1
Amorphous and crystalline interaction coefficient	n	-	1.5	1.6
Shear modulus	$\mu_{B}$	MPa	12	12
Maximum (fully extended) stretch	$\lambda_{\mathrm{lock}}$	-	4	6
Bulk modulus	κ	MPa	500	500
Adjustment coefficient	ξ	-	0.01	0.01
Strain factor	C	-	−0.5	−0.5
Flow shear resistance	${\hat{\tau }}_{B}$	MPa	6	20
Flow shear modulus	m	-	3	3

**Table 3:** Fitting results of PE80 pipe.

c_1_	c_2_	c_3_	c_4_	c_5_	c_6_
0.6474	8.3033	1.2968	−30.6775	1.0973	0.5003

**Table 4:** Fitting results of PE100 pipe.

c_1_	c_2_	c_3_	c_4_	c_5_	c_6_
1.0188	11.9047	1.0376	0.6515	0.9950	0.7924

**Table 5:** Fitting results of PE80 pipe.

A_1_	A_2_	A_3_	A_4_
30.1998	−278.6827	5.8886 × 10^−20^	−9.1673 × 10^−20^

**Table 6:** Fitting results of PE100 pipe.

A_1_	A_2_	A_3_	A_4_
29.1889	9.65381	6.5841 × 10^−20^	3.8583 × 10^−20^

**Table 7:** R2 of the models for PE80.

	Unaged	672 h	1344 h	2352 h	3024 h	10,104 h
Proposed	0.999	0.999	0.999	0.999	0.997	0.996
TNV	0.962	0.984	0.985	0.990	0.990	0.889
BB	0.969	0.984	0.981	0.991	0.992	0.944

**Table 8:** R2 of the models for PE100.

	Unaged	672 h	1344 h	2352 h	3024 h	10,104 h
Proposed	0.982	0.989	0.999	0.999	0.999	0.996
TNV	0.981	0.962	0.951	0.989	0.990	0.991
BB	0.979	0.968	0.977	0.991	0.988	0.992

## Data Availability

Data will be made available on request.

## Appendix A

### Appendix A.1. Eight-Chain Model

The eight-chain (EC) model proposed by Arruda and Boyce is a hyperelastic model that essentially assumes a microscopic structure of elastomers and characterizes their deformation behavior using statistical mechanics [52,59].

The basic assumption of the EC model is that the principal directions of macromolecules (also referred to as chain molecules) are uniformly distributed along the diagonals of a unit cell in the material coordinate system. The edge length of the undeformed unit cell is denoted by $a_{0}$, while the undeformed chain length is represented by $r_{0}$, with the following relationship: $r_{0}=a_{0}\sqrt{3}$. Furthermore, each macromolecule is considered to consist of *n* freely jointed rigid segments of length l. In the absence of external force fields, the mean end-to-end distance is $l\sqrt{n}$. By defining the principal shear stretch ratios ${\tilde{\lambda }}_{1}$, ${\tilde{\lambda }}_{2}$, and ${\tilde{\lambda }}_{3}$, the effective chain length is expressed as:

(A1) $$r=a_{0}[({\tilde{\lambda }}_{1}{)}^{2}+({\tilde{\lambda }}_{2}{)}^{2}+({\tilde{\lambda }}_{2}{)}^{2}{]}^{1/2},$$

The effective chain elongation is:

(A2) $$\bar{\tilde{\lambda }}=[\frac{({\tilde{\lambda }}_{1}{)}^{2}+({\tilde{\lambda }}_{2}{)}^{2}+({\tilde{\lambda }}_{3}{)}^{2}}{3}{]}^{1/2}=\sqrt{\frac{\mathrm{tr}\left(\tilde{C}\right)}{3}}=\sqrt{\frac{\mathrm{tr}\left(\tilde{b}\right)}{3}}=\sqrt{\frac{{\tilde{I}}_{1}}{3}},$$

where $\tilde{b}={\left(J\right)}^{-2/3}b$. This indicates that the shear chain stretch ratio is essentially a function solely of its first invariant. Based on this physically motivated activation model, the eight-chain model is formulated as an isotropic thermoelastic model, where the Helmholtz free energy Ψ per unit reference volume depends exclusively on two invariants of the deformation gradient and temperature: $\tilde{\lambda }\left(\tilde{b}\right)={\left[tr\left(\tilde{b}\right)/3\right]}^{1/2}$, J=det(F), and $\theta_{0}$. The Cauchy stress in the EC model can be derived from continuum mechanics principles as follows:

(A3) $$T=\frac{2}{J}\left[\frac{\partial \Psi }{\partial {\tilde{I}}_{1}}+\frac{\partial \Psi }{\partial {\tilde{I}}_{2}}{\tilde{I}}_{2}\right]\tilde{b}-\frac{2}{J}\frac{\partial \Psi }{\partial {\tilde{I}}_{2}}{\left(\tilde{b}\right)}^{2}+\left[\frac{\partial \Psi }{\partial J}-\frac{2{\tilde{I}}_{1}}{3J}\frac{\partial \Psi }{\partial {\tilde{I}}_{1}}-\frac{4{\tilde{I}}_{2}}{3J}\frac{\partial \Psi }{\partial {\tilde{I}}_{2}}\right],$$

For elastomers, the internal energy is typically not a function of the applied shear stretch ratio. The Helmholtz free energy is generally expressed as:

(A4) $$\Psi \left(\bar{\tilde{\lambda }},J,\theta_{0}\right)=e_{0}(J)-\theta_{0}\eta_{0}\left(\bar{\tilde{\lambda }}\right),$$

Due to the assumption of negligible volumetric changes, the dependence of $\eta_{0}\left(\bar{\tilde{\lambda }},J\right)$ on J is disregarded. The small-strain hypothesis further enables the relationship between Cauchy stress and volumetric deformation to be treated as linear:

(A5) $$T:I=\frac{\partial \Psi \left(\bar{\tilde{\lambda }},J,\theta_{0}\right)}{\partial J}=\kappa (J-1),$$

Given the internal energy $e_{0}(J)=\kappa J(J/2-1)$, the Cauchy stress can be computed according to the governing equation, yielding:

(A6) $$T=\frac{-\theta_{0}}{3J\tilde{\lambda }}\frac{\partial \eta_{0}\left(\tilde{\lambda }\right)}{\partial \tilde{\lambda }}{\tilde{b}}^{\mathrm{dev}}+\kappa [J-1]I,$$

According to the chain rule, the dependence of entropy on the effective chain stretch is determined as follows:

(A7) $$\frac{\partial \eta_{0}\left(r\left(\bar{\tilde{\lambda }}\right)\right)}{\partial \bar{\tilde{\lambda }}}=\frac{\partial \eta_{0}}{\partial r}\frac{\partial r}{\partial \bar{\tilde{\lambda }}}=\frac{\partial \eta_{0}}{\partial r}\frac{1}{\frac{\partial }{\partial r}[rl\sqrt{n}]}=l\sqrt{n}\frac{\partial \eta_{0}}{\partial r},$$

Consequently, the final formulation of the constitutive equation reduces to determining how the entropy of an individual macromolecule depends on its end-to-end distance. The derivation of the Langevin equation (provided below) reveals that:

(A8) $$T=\frac{Nk_{B}\theta }{3J}\frac{\lambda^{\mathrm{lock}}}{\bar{\tilde{\lambda }}}L^{-1}\left(\frac{\bar{\tilde{\lambda }}}{\lambda^{\mathrm{lock}}}\right){\tilde{b}}^{\mathrm{dev}}+\kappa [J-1]I,$$

where $\lambda^{\mathrm{lock}}\equiv nl$ is the maximum elongation for the molecular chain. Bergström provided a numerical approximation for the Langevin function and its inverse function:

(A9) $$L(x)\approx 3x+\frac{9}{5}x^{3}+\frac{297}{175}x^{5}+\frac{1539}{875}x^{7},$$

(A10) $$L^{-1}(x)\approx \left\{\begin{array}{ll} 1.31446\tan(1.58986x)+0.91209x, & \mathrm{if}|\;x\;|<0.84136,\\ 1/(sign(x)-x), & \mathrm{if}\;0.84136\leq |\;x\;|<1 \end{array},\right.$$

### Appendix A.2. Molecular Chain Model

The viscous flow rate of Network B derives from the macromolecular chain “reptation” theory, which conceptualizes polymer-chain environments as confining tubes. Crucially, we hypothesize that molecules with low load-bearing capacity but significant conformational rearrangement during creep govern the time-dependent stress–strain response in polyethylene pipes. Consider initially a simplified single free chain model. Under high-rate network deformation, this chain deforms coordinately, transitioning from a disordered to an ordered state. This entropy-reducing process generates deformation-resisting internal forces. If the strain is subsequently held constant, Brownian motion drives the chain’s gradual recovery toward a relaxed configuration. The recovery kinetics are quantified by reptation theory governing chain dynamics. Although completely free chains are absent in real systems, polymer-network chain ends exhibit analogous behavior. To develop a time-dependent constitutive equation, we focus on chain ends constrained within tubes. Their motion arises from synergistic reptation and contour length fluctuations induced by Brownian motion, resulting in oscillatory displacement along the tube axis. The mean displacement is characterized via the Doi–Edwards reptation theory [66].

### Appendix A.3. Weight Decision

The weight determination process consists of three main steps:

1. Normalization:
  Apply positive range standardization to both crystallinity and carbonyl index to eliminate dimensional effects.
2. Entropy Calculation:
  Compute the information entropy for each normalized parameter.
3. Weight Determination:
  Derive the final weights based on the entropy values.

### Appendix A.4. LM-NMAD

The normalized median absolute deviation (NMAD) is defined as:

(A11) $$\mathrm{NMAD}=\frac{1}{N}\sum_{i=1}^{N}\frac{\left|T-\dot{T}\right|}{median\left|T\right|},$$

The denominator is the median of the absolute values of the observations, which is a constant.

Due to the presence of absolute values, the Huber function is used for the approximate processing of absolute values:

(A12) $$\phi_{\delta }(r)=\delta^{2}\left(\sqrt{1+{\left(r/\delta \right)}^{2}}-1\right),$$

(A13) $$\mathrm{NMAD}=\frac{1}{N\cdot \bar{T}}\sum_{i=1}^{N}\phi_{\delta }\left(r_{i}\right),$$

where $r_{i}=T-\dot{T}$, δ is the parameter that controls smoothness (usually 0.2 times the standard deviation of the experimental data).

Then, to use the LM algorithm, we approximate the NMAD loss of the current iteration as a weighted least-squares problem.

(A14) $$\phi_{\delta }\left(r_{i}\right)\approx \frac{1}{2}w_{i}r_{i}^{2},\mathrm{when}\left|r_{i}\right|\ll \delta \;\mathrm{and}\;\mathrm{NMAD}\approx \frac{1}{N\cdot \bar{T}}\sum_{i}\frac{1}{2}w_{i}r_{i}^{2},$$

1. Initialization
  Given initial parameters: $a^{\left(0\right)}$, initial damping $\lambda^{\left(0\right)}$, smoothing parameter δ, maximum number of iterations: max_iter, convergence tolerance: tol.
  Calculate the median value of the absolute value of the experimental data: $\bar{T}=\;median\left|T\right|$.
2. Iteration steps (for the k-th iteration):
  a. Calculate the model prediction T and the residual $r^{\left(k\right)}=T-\dot{T}$ using the current parameters $a^{\left(k\right)}$.
  b. Calculate weight: $w_{i}^{\left(k\right)}=1/\sqrt{1+{\left(r_{i}^{\left(k\right)}/\delta \right)}^{2}}$
  c. Calculate the objective function value (NMAD): $L^{\left(k\right)}=\frac{1}{N\cdot \bar{T}}\sum_{i}\phi_{\delta }(r_{i}^{\left(k\right)})$
  d. Calculate Jacobian matrix: J, where $J_{ij}=\partial r_{i}/\partial a_{j}$(in $a^{\left(k\right)}$).
  e. Construct a weighted least-squares problem:
    Gradient: $g=J^{⊤}Wr^{\left(k\right)}$, where $W=\mathrm{diag}(w^{\left(k\right)})$.
    Approximate Hessian: $H=J^{⊤}WJ$.
  f. Solving the LM equation:$\left(H+\lambda^{\left(k\right)}\mathrm{diag}\;(H)\right)\delta =-g$, where diag(H) is damping.
  g. Calculate candidate parameters: $a_{\mathrm{candidate}}=a^{\left(k\right)}+\delta$.
  h. Calculate candidate residual and loss: $L_{\mathrm{candidate}}$.
  i. Calculate gain ratio: $\rho =\frac{L^{\left(k\right)}-L_{\mathrm{candidatc}}}{\delta^{⊤}\left(\lambda^{\left(k\right)}diag(H)\delta -\delta^{⊤}g\right)}$
  j. Adjust damping λ according to ρ:
    if ρ>0 (accept): $a^{\left(k+1\right)}=a_{\mathrm{candidate}}$ and $\lambda^{\left(k+1\right)}=\lambda^{\left(k\right)}\max(1/3,1-{\left(2\rho -1\right)}^{3})$
    else: $a^{\left(k+1\right)}=a^{\left(k\right)}$, $\lambda^{\left(k+1\right)}=\lambda^{\left(k\right)}\times \nu$
  k. Check convergence:
    If $\left\|\delta \;\|<\mathrm{tol}\cdot (\|a^{\left(k\right)}\|+\mathrm{tol}\right)$, or the objective function change is less than the tolerance, or when the maximum number of iterations is reached, it stops.
3. Output optimal parameters: $a^{*}$.
